# Energy Requirements in the Post-ICU Period: An Exploratory Multicenter Observational Study

**DOI:** 10.3390/nu17122046

**Published:** 2025-06-19

**Authors:** Marialaura Scarcella, Emidio Scarpellini, Ludovico Abenavoli, Andrea Ceccarelli, Rita Commissari, Riccardo Monti, Jan Tack, Antonella Cotoia, Edoardo De Robertis

**Affiliations:** 1 Anesthesia, Intensive Care and Nutritional Science, Azienda Ospedalier-Universitaria “Santa Maria”, Via Tristano di Joannuccio, 05100 Terni, Italy; m.scarcella@aospterni.it (M.S.); r.commissari@aospterni.it (R.C.); r.monti@aospterni.it (R.M.); 2 Translationeel Onderzoek van Gastro-Enterologische Aandoeningen (T.A.R.G.I.D.), Gasthuisberg University Hospital, KU Leuven, Herestraat 49, 3000 Leuven, Belgium; jan.tack@kuleuven.be; 3 Internal Medicine and Nutrition Unit, “Madonna del Soccorso” General Hospital, 63074 San Benedetto del Tronto, Italy; andrea.ceccarelli@sanita.marche.it; 4 Department of Health Sciences, University “Magna Graecia”, 88100 Catanzaro, Italy; l.abenavoli@unicz.it; 5 Department of Intensive Care, University Hospital of Foggia, 71010 Foggia, Italy; antonella.cotoia@unifg.it; 6 Anesthesia Department, Perugia University, 06121 Perugia, Italy; edoardo.derobertis@unipg.it

**Keywords:** nutritional transition, bioimpedance vectorial analysis, energy expenditure, nutritional assessment, post-critical patient

## Abstract

Background: There is limited knowledge about nutritional intake and energy needs during the post-intensive care unit (ICU) period and their relationship with clinical outcomes and physical recovery. Aims and Methods: Thus, this observational multicenter study (Azienda Ospedaliero-Universitaria “Santa Maria”, Terni and “Madonna del Soccorso” General hospital, San Benedetto del Tronto, Italy) aimed, firstly, to measure energy expenditure via indirect calorimetry (IC) (Q-NRG+^®^ Metabolic Monitor, Cosmed, Rome, Italy), derived respiratory quotient (R/Q1) and, malnutrition risk via Mini Nutritional Assessment (MNA) test and body composition through bioimpedance vector analysis (BIVA-Akern, Pontassieve, Italy); secondly, to assess their effect on energy needs, body composition and physical rehabilitation steps in critically ill adults after ICU discharge. The provision of nutrients (PIS test) was also recorded. Oral nutritional supplementation was used to reach the optimal nutritional intake. All patients followed a standardized rehabilitation program. Results: A total of 43 patients were enrolled from January 2024 until February 2025 at the beginning of their post-ICU period. The mean age was 65.7 ± 1.0 years, the mean BMI was 20.73 ± 0.8 kg/m^2^ at the recovery ward, and 60.4% (*n* = 26) were male. The mean admission period was 19.5 ± 1.7 days. The resting energy expenditure (mREE) was 1591 ± 71.2 at the admission and 1.856 ± 62.7 kcal/kg/d at the discharge (*p* < 0.05). The median phase angle value was 4.33 ± 0.15 at the admission and 5.05 ± 0.17° at the discharge (*p* < 0.05); R/Q1 at the admission was 0.7 ± 0.1 and 1.086± 0.11 at the discharge (*p* < 0.05). Improved energy expenditure significantly correlated with R/Q1 and phase angle (r = 0.81 and r = 0.72, respectively). Interestingly, there was no significant correlation between improved metabolism and improved PIS test scores (r = 0.18). Improved metabolism and nutritional status showed a tendency to correlate with shorter post-ICU courses and earlier physical recovery, without reaching statistical significance. Conclusions: Measurement of energy expenditure and caloric intake, along with the assessment of body composition is feasible and provides an objective tool to guide and possibly enhance the functional recovery in patients during the post-ICU period.

## 1. Introduction

The transition from the intensive care unit (ICU) to mild intensity clinic and eventual discharge is a crucial period in the recovery of critically ill patients. During this timelapse, patients pass through significant physiological and metabolic changes. They include persistent catabolism, muscle wasting, impaired immune function, and reduced physical capability [1]. These issues can retard the rehabilitation process, prolong hospitalization, and increase the risk of hospital readmission [1]. The latter can be complicated by increased mortality and morbidity of patients [2].

One of the key determinants of post-ICU recovery is adequate nutritional support [1,2].

In fact, ICU survivors frequently experience energy and protein waste and loss due to altered metabolism, anorexia/hyporexia, dysphagia, gastro-intestinal dysfunction, and challenges in enteral or oral feeding [3]. These nutritional “gaps” can hinder muscle preservation, compromise immune defense, and impair long-term functional outcomes [4]. Thus, it is crucial to establish nutritional protocols to gather the nutritional/energy needs of post-ICU patients [1,5].

However, despite growing recognition of the importance of nutrition in the post-ICU period, there is limited data on optimal energy and protein intake targets. This is also explained by the lack of “individualized” strategies to assess individual nutritional requirements [5,6]. In this regard, the clinical and nutritional heterogeneity of critically ill patients and fluctuations in post-ICU recovery periods make it difficult to build and validate uniform guidelines. Investigations on the basal metabolic state, its measurement via validated methods (e.g., indirect calorimetry (IC)), and its use to drive personalized nutritional and physical rehabilitation programs are few and heterogeneous [5,6].

To address these gaps, this observational and exploratory study sought, firstly, to measure energy expenditure and body composition in post-ICU patients and, secondly, to assess the effect of these assessments on energy needs, body composition, and physical rehabilitation steps. 

## 2. Materials and Methods

### 2.1. Study Design

This observational, multicenter prospective study followed up on post-ICU discharged patients at “Madonna del Soccorso” General Hospital, San Benedetto del Tronto, and Azienda Ospedaliero-Universitaria “Santa Maria”, Terni, Italy between January 2024 and February 2025. We respected regional Ethical Committee rules for patients’ enrollment (Ethical Committee CEAS Umbria, CER N4757/24, March 2024; Ethical Committee CERM Marche, CER N4146/13, March 2024; Italy). Enrollment was operated upon patients’ informed consent signatures.

Patients were enrolled at the moment of ICU discharge and followed up during their mild-intensity unit admission and hospital stay, until discharge.

**Figure 1:**
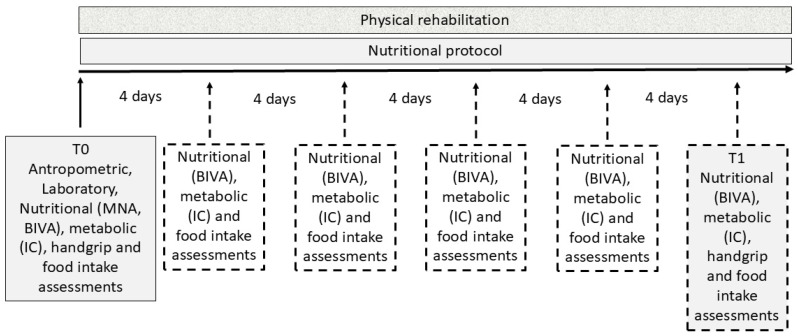
Study design representation. All patients underwent preliminary anthropometric and laboratory tests, nutritional (bioimpedance vector analysis (BIVA)), metabolic (indirect calorimetry (IC)), muscle strength (handgrip), and food intake assessments at admission (namely, T0) and discharge (T1). Mini Nutritional Assessment (MNA) test was taken at mild-intensity clinic entrance. Laboratory test monitoring continued throughout the mild-intensity clinic stay. Nutritional, metabolic, and food intake assessments were taken every four days throughout the patient’s post-ICU stay. All patients followed standardized nutritional and physical rehabilitation protocols.

For each patient, anthropometric data was detected at ICU (T0) and mild-intensity clinic discharge (T1), respectively. Mini Nutritional Assessment (MNA) test was taken at mild-intensity clinic admission. Moreover, metabolic and nutritional, body composition assessments (through indirect calorimetry and bioimpedance vector analysis (BIVA), respectively) were carried out at T0 and at regular intervals (namely, four days) throughout the mild-intensity clinic stay (at least 4 measurements per patient). In addition, each patient was also monitored for the amount of food taken with the meal through the Progressive Intake Scale (PIS) test and possibly supported with oral nutritional supplements (ONS) in case of protein caloric intake < 60% of the daily requirement. The daily oral nutritional scheme according to the IC energy requirements, with 1.3 g of protein/kg target (Figure 1).

Participants’ criteria of inclusion: adult patients (age range between 20 and 90 years) previously admitted to the ICU, with a hospital stay exceeding 72 h, and transferred to mild-intensity clinical ward.

Patients were excluded from the study in case of pregnancy, major gastro-intestinal surgery, malabsorption syndromes, inflammatory bowel disease, gastro-intestinal motility disorders, acute/chronic pancreatitis, immunodepression (e.g., acquired immunodepression syndrome (HIV)), hematologic disease, and cognitive status impairment.

### 2.2. Mini Nutritional Assessment (MNA) Test

The Mini Nutritional Assessment test is a multidimensional screening tool, validated in several clinical settings. More specifically, it is an integrated nutrition index that evaluates different nutritional parameters “to obtain a synthetic information and a more accurate nutritional diagnosis” [7]. MNA has 96% sensitivity, 98% specificity, and 97% predictive value to describe the nutritional status of patients [7].

MNA is useful both for the first and follow-up assessment of the nutritional status of elderly patients. Interestingly, when older patients are hospitalized, MNA scores are able to estimate healthcare costs, length of stay, and short-term and long-term mortality. In fact, the test scores have an inverse correlation with these hospitalization items.

More interestingly, the MNA test generates an index of both muscle disability and motility and, in parallel, the nutritional status assessment of hospitalized and non-hospitalized patients [7].

The test is composed of 18 items. These are grouped into three sections: one evaluating anthropometric measurements and weight changes; one estimating the amount and composition of ingested food; and one assessing disabilities and cognitive status [8].

MNA testing encompasses two steps:

- Screening (maximum score of 14, extracted from six variables): Information regarding weight loss over the previous three months, food intake, motility, acute stress, cognitive status, and Body Mass Index (BMI) assessment. In detail, scores between 0 and 7 are predictive of malnutrition, scores between 8 and 11 suggest the risk of malnutrition, and scores between 12 and 14 indicate sufficient nourishment.

In particular, for scores lower than 11, it is strongly recommended that the remaining test items continue to be collected.

For scores higher than 24, the patient is clearly well-nourished. On the other hand, the “grey zone” of scores between 17 and 23.5 suggests a risk of malnutrition.

Finally, when patients score less than 17, they are clearly malnourished.

- Self-Global Assessment (namely, evidence of history of drug use, food habits, and fluid intake assessment, evaluation of place of residence, and scoring of patient’s considerations of personal health status and nutritional status).

### 2.3. Indirect Calorimetry: Resting Energy Expenditure and Respiratory Quotient

Indirect calorimetry measurement has been validated both in laboratory and clinical settings, offering reliable measurements in both ventilated and non-ventilated patients ensuring the possibility of assessing the metabolic status of the patient, both in the critical and the mild-intensity clinic setup.

The resting energy expenditure (mREE) data were obtained through Q-NRG+^®^ Metabolic Monitor (Cosmed, Rome, Italy). The data met steady-state conditions defined by a variance of volume of oxygen (V02) and volume of carbon dioxide (VC02) of <10% as per published validation data for the Q-NRG device [9]. The data were averaged over four-day intervals for patients with multiple IC measurements.

The Respiratory Quotient (RQ) data was collected at the same time interval. RQ is the ratio between carbon dioxide (CO_2_) production and oxygen (O_2_) consumption (namely, R/Q1). This indicates whether the body is primarily metabolizing carbohydrates, fats, or proteins for energy. Since different macronutrients undergo distinct oxidative pathways, their associated RQ values vary. For example, in case of carbohydrate oxidation, the RQ value is around 1.0 because full oxidation of glucose leads to equal CO_2_ production and O_2_ consumption. Further, for proteins, the RQ value is between 0.8 and 0.85. In fact, protein metabolism results in slightly lower CO_2_ output relative to O_2_ uptake. Lipids’ RQ value is between 0.66 and 0.75 because fat oxidation requires more oxygen relative to CO_2_ production [10].

Interestingly, interpreting RQ can provide insight into metabolic balance, nutritional adequacy, and physiological adaptations to disease or dietary intake. In this frame, RQ lower than 0.70 suggests prolonged fasting, underfeeding, or reliance on fat oxidation [8]. These are common features of malnutrition, catabolic states, and of subjects on ketogenic diet. Moreover, the RQ values can describe excessive lipolysis (e.g., uncontrolled diabetes, starvation, or prolonged critical illness).

For extremely low RQ values (≤0.65) measurement protocol mistakes, air leaks in ventilated patients, or metabolic acidosis should be suspected. Oppositely, a high RQ value (>0.90) suggests high carbohydrate intake and, perhaps, overfeeding. Similarly, an RQ exceeding 1.0 suggests lipogenesis (namely, fat storage) when the body is converting excess carbohydrates into fat. This results in increased CO_2_ production and threatens outcome of ventilation-dependent patients.

Indeed, normal RQ value (range between 0.75 and 0.85) normally describes balanced energy intake, with a mix of fat, carbohydrate, and protein metabolism, typically retrieved in well-nourished patients [11].

Beyond its role in guiding nutritional therapy, RQ can serve as a marker of metabolic adaptation in various clinical conditions.

Thus, RQ serves as a key indicator of overfeeding/underfeeding and reveals the body’s ability to utilize different nutrients. Variations in RQ highlight metabolic shifts, indicating inadequate or excessive feeding. However, careful interpretation of RQ values is essential, considering confounding factors (e.g., disease state, feeding regimen).

### 2.4. Bioimpedance Vector Analysis

Bioelectrical impedance analysis (BIA) is a non-invasive tool to assess human body composition (e.g., fat, bone, water, and muscle content). The tool delivers a low-frequency electrical current according to the principle that fluid and cellular structures present different levels of resistance to the current passing through a living system. BIA measurements are resistance (R-Ohms), describing cellular hydration; reactance (Xc—Ohms), describing tissue integrity, and phase angle (PhA—degrees), which is the arc tangent between R and Xc. Thus, BIA evaluates hydration and nutrition in men [12].

Bioelectrical impedance vector analysis (BIVA) assesses body composition in patients with advanced illnesses, such as those admitted to intensive care. In fact, statistical vector analysis of BIA data better describes body composition in this subset of patients [12]. In detail, BIVA uses graphical vectors to analyze BIA data. Thus, impedance (Z) is plotted as a vector from its components R (*X*-axis) and Xc (*Y*-axis), after being standardized by height (H). The RXc graph represents the sex- and race-specific tolerance intervals of a comparative reference population. Tolerance ellipses are plotted on the RXc graph to represent the 50%, 75%, and 95% centiles (i.e., confidence intervals) for the population in the study. This method allows a simultaneous assessment of tissue hydration or soft tissue mass changes independent of regression equations or body weight. For these reasons, BIVA can also be interpreted accurately in critically ill ICU patients at extremes of weight or volume distribution.

Patients were evaluated with BIA 101 BIVA (Akern, Pontassieve, Italy) equipment.

### 2.5. Energy and Protein Intake Evaluation

The Progressive Intake Scale (PIS) is a population-based validated observational tool commonly used in clinical settings, especially in hospital wards, to monitor and estimate caloric and protein intake [13,14]. Its validity has been extensively and systematically reviewed [14]. The Tool provides real-time monitoring of nutritional intake (energy and protein) in inpatients. In detail, it can detect early signs of nutritional risk or malnutrition, offering objective data supporting intervention of the nutritional team. Practically, the PIS allows one to observe and quantify food consumption during meals, providing a visual estimate of the amount of food eaten (e.g., 25% of the presented dish, 50% of the presented dish).

In further detail, PIS uses a 5-point scale to represent the percentage of food consumed by the patient:


**Score**

**Description**

**Estimated % of Meal Consumed**
0No intake0%1Poor intake~25%2Moderate intake~50%3Good intake~75%4Full intake100%

### 2.6. Muscle Strength Assessment

A trained dietitian used handgrip dynamometry (Jamar for Cosmed, Rome, Italy) to assess upper limb muscle strength bilaterally [15]. Patients were assessed in a seated position, in a chair where possible, or sitting at least at 45° in bed, with their forearm in flexion at 90° and wrist in extension, supported by the arm of the chair or a pillow. Patients were asked to perform a maximal voluntary isometric contraction of their dominant hand and, maintain the contraction for 3–5 s. Three consecutive isometric contractions with 30–60 s of rest in between tests were completed and the highest measurement was recorded. Strength was expressed as kg.

### 2.7. Statistical Analysis

Statistical analyses were performed using SPSS Software 21 (IBM, New York, NY, USA). Quantitative variables distribution was tested with the Kolmogorov–Smirnov normality test. All data are presented as mean ± standard deviation (SD) or median [inter-quartile range, IQR] according to the normal or not normal distribution. Parametric (Student’s *t*-test) and non-parametric tests (Mann–Whitney U test) were applied to describe the differences between groups for the variables of interest as appropriate. Analysis of variance was performed to assess variable modifications over time, with correction for multiple tests. The magnitude of the interventional effect was evaluated by Cohen’s d test for paired samples. The alpha level of significance was set at 0.05. The sample size was calculated to measure a difference of measured variables of at least 30%. Bonferroni correction for errors due to multiple testing was applied when needed [16].

## 3. Results

A total of 43 patients were enrolled from 1 January 2024 until the end of February 2025 from the Internal Medicine mild-intensity clinic of Azienda Ospedaliero-Universitaria “Santa Maria”, Terni and Internal Medicine Unit of “Madonna del Soccorso” General Hospital, San Benedetto, Italy.

The mean age was 65.7 ± 1.0 years, and the mean BMI was 20.73 ± 0.8 kg/m^2^ at the admission to the mild-intensity clinic (none of the participants had a BMI below 20.0 kg/m^2^); 60.4% (*n* = 26) of participants were male.

The main comorbidities were diabetes (23%, type 2.84%), hypertension (42%), chronic ischemic heart disease (26%), chronic obstructive pulmonary disease (COPD) (28%), anxiety, and depression (8%).

The main causes of ICU admission were major trauma (*n* = 8), septic shock (*n* = 11), multiorgan failure (MOF) (*n* = 6), and respiratory failure (*n* = 20).

The average hospital stay duration was 48.2 ± 3.7 days, with an ICU stay averaging 28.7 ± 2.7 days.

There were no significant gender differences in most population characteristics. Obesity was more prevalent among females vs. men (*p* < 0.05).

MNA test values significantly correlated with BIVA Pha values (Table 1).

The median measured REE was 1591 ± 71.2 kcal/kg/d at the admission to the ward. Accordingly, the R/Q1 ratio at the admission to the mild-intensity clinic was 0.7 ± 0.1.

The phase angle value was a median of 4.33 ± 0.15° at the discharge from the ICU.

**Figure 2:**
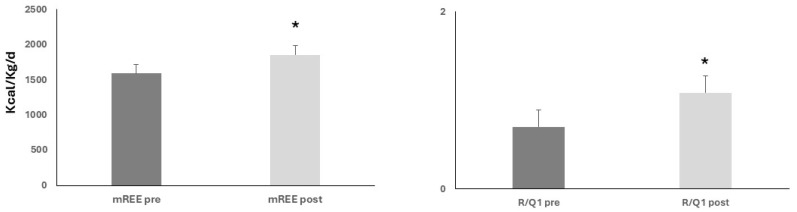
Upon nutritional and rehabilitation interventions, baseline resting energy expenditure (mREE) and respiratory quotient (R/Q1) showed a significant rise; ANOVA, both * *p* < 0.05.

Upon nutritional and physical rehabilitation interventions (19.5 ± 1.7 days), mREE showed a significant rise (1591 ± 71.2 vs. 1.856 ± 62.7 kcal/kg/d, ANOVA, *p* < 0.05). Similarly, the R/Q1 ratio showed a similar rise from baseline (0.7 ± 0.1 and 1.086 ± 0.11, ANOVA, *p* < 0.05) (Figure 2).

**Figure 3:**
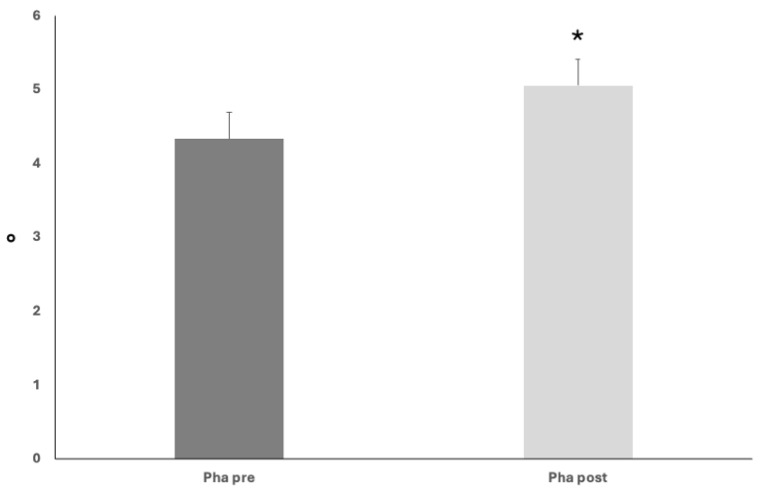
Upon nutritional and rehabilitation interventions, baseline phase angle (Pha) showed a significant rise; ANOVA, * *p* < 0.05.

Pha values significantly rose from baseline until mild-intensity clinic discharge (4.33 ± 0.15 vs. 5.05 ± 0.17°, ANOVA, *p* < 0.05) (Figure 3).

**Figure 4:**
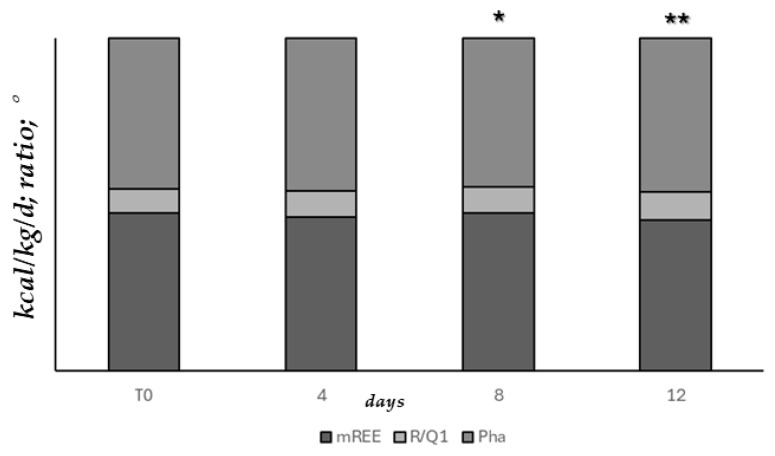
Basal metabolism and nutritional status measurements assessed since T0 and during mild-intensity clinic stay, at 4-day intervals (ANOVA, * T0 vs. 8 days upon admission *p* = 0.058, ** T0 vs. 12 days upon admission, *p* < 0.05).

Figure 4 represents the consensual temporal improvement of both basal metabolism (mREE, R/Q1) and nutritional status (Pha) measurements assessed since T0 and during the mild-intensity clinic stay of patients at 4-day intervals (ANOVA, T0 vs. 8 days upon admission *p* = 0.058, ** T0 vs. 12 days upon admission, *p* < 0.05).

Handgrip performance significantly rose from admission (23 ± 1.2 kg) to mild-intensity unit discharge (29.5 ± 1.1 kg), ANOVA, *p* < 0.05. Four patients (two for each enrolling hospital) were unable to perform the measurement because of arm movement limitations.

**Figure 5:**
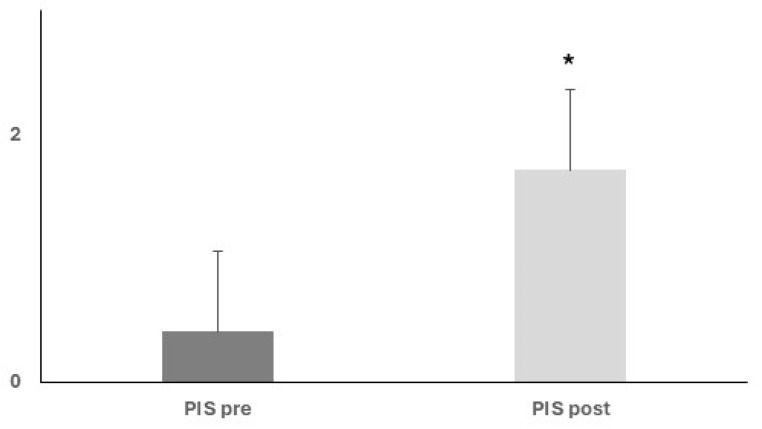
Upon nutritional and rehabilitation interventions, baseline PIS score showed a progressive and significant rise; ANOVA, * *p* < 0.05.

PIS scores progressively and significantly rose from baseline to mild-intensity clinic discharge (0.41 ± 0.1 vs. 1.71 ± 0.18, ANOVA, *p* < 0.05) (Figure 5).

Cohen’s d test showed significant d values (precisely, for mREE, d = −1.45; for Pha, d = −1.59; for PIS, d = −2.16), indicating a significant clinical impact of the nutritional and physical rehabilitation interventions performed.

Twenty-four % of patients were reported as having pre-ICU existing oropharyngeal dysphagia (OPD) that remained stable throughout the mild-intensity clinic stay and at discharge (precisely, 22%), *p* = NS.

There were no statistical differences between ONS- and non-ONS-administered patients for all the nutritional and metabolic variables measured (all, *p* = NS).

**Figure 6:**
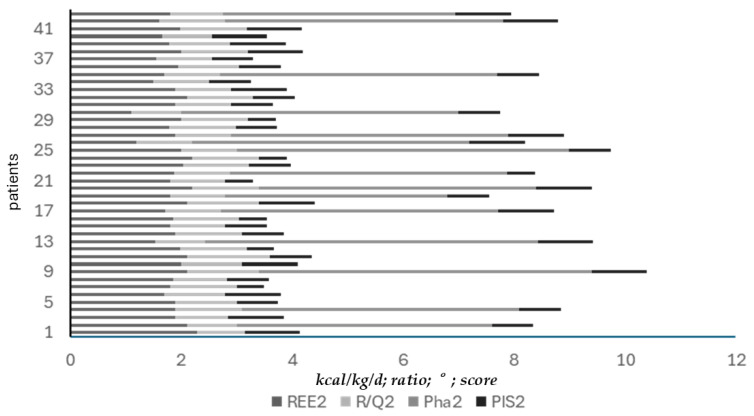
Correlation between mREE and R/Q1, Pha, and PIS scores at mild-intensity clinic discharge. Improved mREE values significantly correlated with improved R/Q1 (r = 0.81). Improved metabolism (mREE) significantly correlated with improved nutritional status (Pha) (r = 0.72). However, improved metabolism (mREE) did not follow the rise of PIS score.

Interestingly, while the rise of mREE significantly correlated with those of R/Q1 and, Pha (respectively, r = 0.81 and r = 0.72), it did not reach statistical significance for PIS scores (r = 0.18) (Figure 6).

Improved metabolism and nutritional status showed a tendency to have an inverse correlation with the shorter post-ICU course and earlier physical recovery, without reaching statistical significance (r = −0.23 and r = −0.26, for energy expenditure; r = −0.24 and r = −0.28, for BIVA, respectively).

Four patients had septic complications and two suffered from respiratory failure, requiring ICU readmission. Of importance, their nutritional status and basal metabolism were significantly lower than mild-intensity clinic successfully discharged patients (Pha: 4.21 ± 0.13 vs. 5.07 ± 0.16°, for ICU readmitted vs. non-ICU readmitted patients, respectively, *p* < 0.05; mREE: 1527 ± 65.1 vs. 1.868 ± 62.1 kcal/kg/d, for ICU readmitted vs. non-ICU readmitted patients, respectively, *p* < 0.05; R/Q1: 0.65 ± 0.2 and 1.087 ± 0.12, for ICU readmitted vs. non-ICU readmitted patients, respectively, *p* < 0.05).

To note, metabolic and nutritional parameters measured at mild-intensity clinic discharge (namely, T1) did not differ significantly between the two enrolling hospitals (all, *p* = NS).

## 4. Discussion

Results from the investigation showed that the combined measurement of energy expenditure and caloric intake, together with the assessment of body composition, is useful for monitoring the nutritional, metabolic, and physical recovery of ICU-discharged patients. The latter, perhaps, has been described by the improved muscle strength measurement via handgrip. Interestingly, there was not a strong correlation between improved nutritional and metabolic status and the amount of ingested food, as described by the PIS score. Finally, improved metabolism and nutritional status showed a tendency to have an inverse correlation with shorter post-ICU courses and earlier physical recovery, without reaching statistical significance.

In the literature, there is agreement on the extreme importance of timing and organization of nutritional interventions administered after the acute phase of critical illness [17,18]. Moreover, with the time of ICU permanence and mechanical ventilation endurance, it is difficult to build up and apply a precise nutritional and rehabilitation protocol for the post-critical patient [16,17]. Therefore, quantifying nutritional intake and nutrition-related outcomes after ICU discharge is affected by several issues: equipment and personnel availability, the accurate quantification of volitional ingested nutrient intake, and timing of hospital discharge [19].

The critical patient suffers from an acute inflammatory response and pronounced stress metabolism. These result in increased catabolism, insulin resistance, and “anabolic resistance” [20]. Upregulated production of pro-inflammatory cytokines and mediators is associated with increased muscle catabolism resulting in a net loss of lean body mass, and reduced functional capacity. Thus, the post-ICU patient is often malnourished and sarcopenic [19]. Data from our study confirms a significant number of malnourished patients according to initial BIVA and MNA screening assessments. There were no differences between the two hospitals also (namely, secondary and primary healthcare centers). This finding shows the homogeneity of the population in the study. These percentages agree with other literature studies that describe the prevalence of malnutrition and sarcopenia in post-ICU patients. In detail, the perspective report by Wittholz et al. [6] assessed malnutrition and muscle strength in post-trauma ICU patients until hospital discharge. The authors showed the significant gap between the nutrients and caloric requirements of patients and their intake. The malnutrition incidence was significantly correlated with lower handgrip performance and quadriceps muscle layer thickness. However, this report did not evaluate body composition with BIVA and basal metabolism with IC. In fact, the Subjective Global Assessment (SGA) was used to assess nutritional status and estimated nutritional requirements were calculated using actual body weight for patients below or within the ideal body weight (IBW) range. Both of these are indirect methods lacking the same accuracy as BIVA and IC. Nakamura et al. performed a prospective post-ICU COVID-19 patient follow-up study. In the study, the patients reported a lesser incidence of cognitive post-intensive care syndrome when supplemental parenteral nutrition was used [21]. Again, in the study, nutritional status and basal metabolism were not assessed. Very often, reports on post-ICU patients lack objective measurements of the nutritional status and basal metabolism of post-critical patients, during their rehabilitation phase [22]. Some reports are also biased by the retrospective design of the investigation [23].

Another perspective study by Moonen et al. evaluated the IC-measured basal metabolism of ICU patients also during their post-ICU stay. Results showed that the mean energy requirements during the post-ICU period are higher than those in the ICU. Indeed, data on nutritional status is missing [10]. Ridley et al. showed a similar bias in measurements [19].

Thus, we can conclude that our study shows a complete objective nutritional and metabolic patient assessment during the post-ICU stay. Thus, results can be considered accurate and, more importantly, reproducible. A few reports from the literature used a combined BIVA and muscle assessment of malnutrition and sarcopenia in ICU patients. Indeed, the findings support our results. In a recent report on COVID-19 patients, these assessments showed moderate-to-high correlation with other morpho-functional parameters and good performance in predicting severe malnutrition and complications during ICU admission [24]. Thus, we can conclude that the combined BIVA and muscle ultrasound use can monitor and guide ICU and, also post-ICU patient rehabilitation courses, considering this time-lapse a continuum [25].

About 30% of female patients showed obesity. Apparently, this finding did not affect metabolic and nutritional status improvement after a mild-intensity clinic stay. Main comorbidities did not affect the homogeneity of the enrolled population either. Further, we did not encounter compliance issues due to the study protocol. The number of ICU readmissions is in line with other reports from the literature [26].

In our study, the improvement and rise of basal metabolism (described by mREE and R/Q1) followed those of nutritional status, described by improved Pha. This finding is of importance because there was a significant improvement in free fat mass, as described by BIVA. Therefore, we can hypothesize a significant benefit from the measurements to monitor patients’ metabolic and nutritional recovery. This finding is only in partial agreement with existing literature. In fact, Kvåle et al. reported that 40% of 136 post-ICU patients lost more than 10 kg during a mild-intensity clinic stay. In addition, 6 months after ICU discharge, 35% of patients did not gain weight. Finally, 15% had lost further weight [27]. Thus, pre-illness weight gain can be wrongly interpreted as a positive sign in recovery. Indeed, several studies have reported the weight gain recorded during the rehabilitation period to be due to an increase in fat deposition rather than an increase in muscle mass [28,29]. The current issues on nutrition recovery and rehabilitation after critical illness are supported also by review observations from 16 studies [30].

In our assessment, about 24% of patients reported having pre-ICU existing oropharyngeal dysphagia (OPD). This finding is in line with the literature and is another major burden for ICU survivors to overcome ICU-acquired weakness [31]. Literature data report up to 84% of patients with post-extubation OPD (range 11 to 83%) [27]. Clearly, OPD is associated with malnutrition, prolonged hospital stays, and increased mortality [32]. Dietary approaches to treat OPD are based on modified texture and fluid diets (e.g., puréed food and thickened liquids). The latter can be often associated with reduced calorie and protein intake and a higher risk of worsening pre-existing malnutrition [26]. Finally, the time between extubation and initiation of oral diet is longer in patients with OPD vs. non-dysphagic ones [26]. Looking at patients from the present study, dysphagia prevalence did not change significantly between post-ICU discharge and after mild-intensity unit stay, remaining stable at 22% of patients at the end of the study (ANOVA, *p* = NS). Neither did we record a significant difference between prevalence among the two hospitals’ populations both at enrollment and discharge (24 vs. 22.8% and 22 vs. 21.8%, respectively, both *p* = NS).

ICU-associated muscular weakness is also associated with reduced ability or complete loss of volitional feeding and reduced dietary intake [26]. Herridge et al. enrolled a cohort of ICU patients (about 30% with more than 44 years of age) and found a significant correlation between loss of autonomous feeding capability and more than two weeks of ICU stay. This issue persisted for more than 6 months after ICU discharge [33]. Muscular weakness was confirmed through handgrip assessment at ICU discharge in our study. Moreover, it significantly improved with a progressive increase throughout the study. In other studies, muscle strength was measured with ultrasound (namely, rectus femoris or quadriceps pennation angle), that can be biased by critical patients’ tissue edema but remains one of the gold standard measurements [6,23].

The lack of correlation between improved metabolism and nutritional status and the amount of ingested food (namely, PIS score) can be explained by different factors affecting food intake. In fact, psychological issues, hunger, appetite, and dysphagia grading can significantly affect post-ICU feeding [26,29]. However, muscle weakness also can affect food ingestion capability. Thus, the improvement of metabolism and nutritional status does not invariably imply an improved capability of oral nutrition. Thus, the lack of correlation between improved metabolic and nutritional status of the patients is not surprising, especially in the case of one-fifth of patients with persisting dysphagia and, perhaps muscle weakness.

Data on the efficacy of oral feeding in post-critical patients from the present study is not in complete agreement with the literature. In fact, measuring or calculating oral calorie/protein intake in various post-ICU patient populations has been performed in several studies [34,35]. Some studies have demonstrated that patients relying on an oral diet alone usually ingest 55–75% of prescribed calories and 27–74% of prescribed protein [36]. However, this data relies only on the early phases of mild-intensity clinic recovery. Our study followed up patients for about three weeks after ICU discharge. Thus, it is conceivable that improved muscle functioning and general patient conditions can explain such differences [26,33].

Moreover, in our study, the impact of supplemental enteral/parenteral feeding on the metabolic and nutritional status of patients was not significant. Observations from literature studies seem to confirm that patients who continue to receive enteral nutrition, with or without an oral diet, can reach 62–104% of prescribed calories and 59–100% of prescribed protein load, respectively [32]. However, in our investigation, the follow-up period was longer than the reported study and a paucity of patients required enteral/parenteral nutrition support. The latter can explain the lack of significant impact of these nutritional approaches on the measured parameters. In line with these findings, we can explain the lack of observed impact of ONS used in a paucity of our patients to improve metabolic and nutritional status. Indeed, data from the literature does not report a significant impact of ONS on the nutritional status of post-ICU patients [26].

Finally, due to the small sample size and the relatively short follow-up timing, we did not observe a significant inverse correlation between improved metabolism and nutritional status, shorter post-ICU course, and earlier physical recovery.

The study has several limitations. First, the small sample size of enrolled subjects. Indeed, the sample size was able to detect and describe significant differences in measured parameters throughout the study. Moreover, the difficulties enrolling patients after ICU discharge and requiring an accurate nutritional and rehabilitation protocol can have conditioned the size of the sample.

Second, the study is multicentric from a secondary and primary hospital, respectively. Although we could expect significant differences in patients’ characteristics from the hospitals, we clearly showed that the enrolled patients were similar in terms of anthropometric, metabolic, and nutritional features.

Third, although we measured muscle strength with a handgrip dynamometer, we did not have muscle ultrasound evaluating its thickness (namely, pennation angle). The latter is the gold standard for evaluating muscle trophism in ICU and post-ICU patients [37].

Fourth, the small sample size and the paucity of patients receiving enteral/parenteral or ONS supplementation did not allow us to draw conclusions on the impact of these approaches on the metabolic and nutritional status of patients. Future larger studies are awarded to assess these items, also according to their different composition in micro- and macronutrients.

## 5. Conclusions

The study results show that a combined metabolic and nutritional assessment in both a primary and secondary healthcare center. Moreover, this combined approach is crucial to setting up a complete nutritional and physical rehabilitation protocol in the post-ICU patient. This correlates with a significant improvement in muscle strength. Thus, an organized nutritional and physical rehabilitation protocol based on trained personnel and availability of equipment is needed.

Further, larger and multicentric, perhaps nationwide studies are needed to confirm the results.

## Figures and Tables

**Table 1:** Provides an overview of the demographic, anthropometric, nutritional, and metabolic baseline characteristics of patients.

	Patients (*n*)	AOUT	SGH	*p*-Value
Patients (*n*)	43 (26 males)	20 (11)	23 (12)	NS
Age (years)	65.7 ± 1.0	66.8 ± 0.8	66.4 ± 0.8	NS
BMI (kg/m^2^)	20.73 ± 0.8	20.2 ± 0.9	20.7 ± 0.7	NS
SOFA II score	53.4 ± 8.2	53.5 ± 7.9	54.1 ± 8.0	NS
MNA test (WN/MN/OV (n)	9/23/11	4/12/6	5/11/5	NS
IC (mRee, kcal/kg/d)	1591 ± 71.2	1583 ± 69.7	1594 ± 69.1	NS
R/Q1	0.7 ± 0.1	0.68 ± 0.1	0.71 ± 0.1	NS
BIVA (PhA, °)	4.33 ± 0.15	4.31 ± 0.12	4.34 ± 0.18	NS
PIS score	0.41 ± 0.1	0.39 ± 0.1	0.41 ± 0.12	NS

Table legend: BMI: body mass index; MNA: mini nutritional assessment tests’ percentages of patients being WN: well-nourished, MN: malnourished, OV: overweight; IC: indirect calorimetry; R/Q1: respiratory quotient; BIVA: bioimpedance vector analysis; PIS: progressive intake scale; NS: non-significant. The reported demographic, anthropometric, nutritional, and metabolic parameters from patients enrolled from Azienda Ospedaliero-Universitaria, Terni (AOUT), and San Benedetto General Hospital (SGH), respectively, showed homogeneity of values.

## Data Availability

The data reported in the article are stored in hospital databases secured for privacy reasons.
